# Supplementary material for: Photo-modulated activation of organic bases enabling microencapsulation and on-demand reactivity
Source: Nat Commun. 2024 Mar 30;15:2771. doi: 10.1038/s41467-024-47175-x (PMC10980803; doi:10.1038/s41467-024-47175-x)
Supplement: Supplementary file 1 — Supplementary Information [file 41467_2024_47175_MOESM1_ESM.pdf]

Supplementary Information

Photo-Modulated Activation of Organic Bases Enabling Microencapsulation and On-Demand Reactivity

Wenle Li[1,†,*], Xiaocun Lu[2,†], Jacob M. Diamond[3], Chengtian Shen[4], Bo Jiang,[1] Shi Sun,[1] Jeffrey S. Moore[4] & Nancy R. Sottos[3,*]

[1]School of Materials Science and Engineering, China University of Petroleum (East China), Qingdao, Shandong 266580, China

[2]Department of Chemistry and Biomolecular Science, Clarkson University, Potsdam, NY 13699, United States

[3]Department of Materials Science and Engineering, Beckman Institute for Advanced Science and Technology, University of Illinois at Urbana-Champaign, Urbana, IL 61801, United States

[4]Department of Chemistry, Beckman Institute for Advanced Science and Technology, University of Illinois at Urbana-Champaign, Urbana, IL 61801, United States

[†]These authors contributed equally to this work.

[*]Correspondence to: wli@upc.edu.cn, n-sottos@illinois.edu

Table of Contents

Supplementary Methods 2

Supplementary Figures 5

Supplementary Tables 21

Supplementary Reference 21

Supplementary Methods

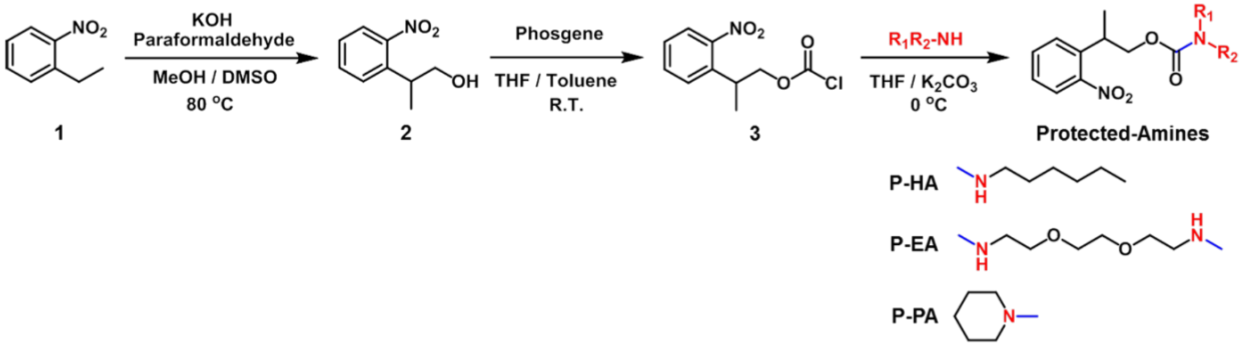

Supplementary Fig. 1 | Synthetic route of NPPOC-protected amines.

The following synthetic route was developed based on a modified procedure.[1]

2-(2-Nitrophenyl)propan-1-ol (**2**). KOH (25.0 g, 446 mmol) was gradually added to the mixture of paraformaldehyde (12.5 g, 416 mmol) and 1-ethyl-2-nitrobenzene **1** (25.0 g, 165 mmol) in 100 mL MeOH/DMSO solvent (1:1, v/v) under N_2 and the reaction mixture was stirring at 70 ºC for 24 h. After cooling to room temperature, the reaction mixture was concentrated *in vacuo*, neutralized with 1 N HCl (300 mL), and extracted with ethyl acetate (3 × 150 mL). After drying over anhydrous $MgSO_4$, the combined organic layers were filtered and concentrated under reduced pressure to give a brownish residue, which was purified by flash column chromatography (SiO_2) eluting with mixed solvent of hexanes and ethyl acetate (hexanes : EtOAc = 5:1 v/v) to afford the titled compound **2** as yellow oil: 17.9 g (60%). ^{1}H NMR (500 MHz, $CDCl_3$, ppm): δ 7.75 (dd, *J* = 8.0, 1.5 Hz, 1H), 7.58 (ddd, *J* = 8.0, 6.5, 1.5 Hz, 1H), 7.50 (dd, *J* = 8.0, 1.5 Hz, 1H), 7.36 (ddd, *J* = 8.0, 6.5, 1.5 Hz, 1H), 3.83 – 3.72 (m, 2H), 3.56 – 3.45 (m, 1H), 1.33 (d, *J* = 7.0 Hz, 3H); ^{13}C NMR (126 MHz, $CDCl_3$, ppm) δ 150.6, 138.1, 132.4, 128.1, 127.1, 124.0, 67.7, 36.3, 17.5.

2-(2-Nitrophenyl)propyl chloroformate (**NPPOC-Cl, 3**). Compound **2** (5 g, 28 mmol) was dissolved in anhydrous THF (10 mL) and cooled to 0 °C. Phosgene solution (15% in toluene, 30 mL, 42 mmol) was then slowly added over a period of 15 min under N_2. After 30 min, the ice bath was removed and the reaction mixture was kept stirring at room temperature for 2 h. Then the reaction solution was slowly bubbled with N_2 for 1 h to remove excess phosgene, followed by complete evaporation of the solvent *in vacuo* to give the titled compound **3** as brown oil: 6.9 g. Compound **3** was directly used for the next reaction without further purification.

NPPOC-protected hexylamine (**P-HA**). Compound **3** (6.9 g, 28 mmol) was mixed with 20 mL anhydrous THF and added dropwise to the mixture of hexylamine (2.8 g, 28 mmol), K_2CO_3 (7.7 g, 56 mmol) and anhydrous THF (40 mL) under N_2 at 0 °C. After vigorous stirring for 30 min, the ice bath was removed and the reaction mixture was kept stirring at room temperature for another 12 h. The reaction mixture was concentrated under the reduced pressure and redissolved in ethyl acetate (50 mL), followed by thorough washing with brine (5 × 50 mL). After drying over anhydrous $MgSO_4$, the organic layer were filtered and concentrated under reduced pressure to give a yellow residue, which was purified by flash column chromatography (SiO_2) eluting with mixed solvent of hexanes and ethyl acetate (hexanes : EtOAc = 3:1 v/v) to afford the titled compound **P-HA** as sticky yellow oil: 7.0 g (81%). ^{1}H NMR (500 MHz, d-DMSO, ppm): δ 7.81 (d, *J* = 8.5 Hz, 1H), 7.69 – 7.66 (m, 2H), 7.50 – 7.45 (m, 1H), 6.99 (t, *J* = 5.5 Hz, 1H), 4.17 – 4.09 (m, 2H), 3.44 – 3.38 (m, 1H), 2.95 – 2.85 (m, 2H), 1.34 – 1.30 (m, 2H), 1.25 (d, *J* = 7.0 Hz, 3H), 1.22 – 1.16 (m, 6H), 0.84 (t, *J* = 7.0 Hz, 3H); ^{13}C NMR (126 MHz, d-DMSO, ppm) δ 155.89, 150.12, 136.93, 132.83, 128.49, 127.65, 123.50, 67.70, 40.61, 33.16, 30.86, 25.84, 25.73, 22.16, 18.01, 14.06. ESI-MS (m/z): $[M+Na]^+$ monoisotopic peak for C16H24N2O4Na, calculated: 331.1634, found: 331.1628; The isotopic signature observed for this compound also confirmed its elemental composition.

NPPOC-protected 2,2'-(ethylenedioxy)bis(ethylamine) (**P-EA**). Compound **3** (6.9 g, 28 mmol) was mixed with 20 mL anhydrous THF and added dropwise to the mixture of 2,2'-(ethylenedioxy)bis(ethylamine) (2.1 g, 14 mmol), K_2CO_3 (7.7 g, 56 mmol) and anhydrous THF (40 mL) under N_2 at 0 °C. After vigorous stirring for 30 min, the ice bath was removed and the reaction mixture was kept stirring at room temperature for another 12 h. The reaction mixture was concentrated under the reduced pressure and redissolved in ethyl acetate (50 mL), followed by thorough washing with brine (5 × 50 mL). After drying over anhydrous $MgSO_4$, the organic layer were filtered and concentrated under reduced pressure to give a yellow residue, which was purified by flash column chromatography (SiO_2) eluting with mixed solvent of hexanes and ethyl acetate (hexanes : EtOAc = 1:1 v/v) to afford the titled compound **P-EA** as sticky yellow oil: 6.7 g (85%). ^{1}H NMR (500 MHz, d-DMSO, ppm): δ 7.81 (d, *J* = 8.0 Hz, 2H), 7.70 – 7.62 (m, 4H), 7.49 – 7.44 (m, 2H), 7.04 (t, *J* = 5.5 Hz, 2H), 4.18 – 4.10 (m, 4H), 3.45 (s, 4H), 3.44 – 3.40 (m, 2H), 3.34 (t, *J* = 6.0 Hz, 4H), 3.11 – 3.03 (m, 4H), 1.26 (d, *J* = 7.0 Hz, 6H); ^{13}C NMR (126 MHz, d-DMSO,

ppm) δ 155.99, 150.10, 136.90, 132.86, 128.52, 127.67, 123.71, 69.47, 69.03, 67.40, 40.48, 33.34, 17.98. ESI-MS (m/z): $[M+H]^+$ monoisotopic peak for C26H34N4O10, calculated: 563.2353, found: 563.2354; The isotopic signature observed for this compound also confirmed its elemental composition.

NPPOC-protected piperidine (**P-PA**). Compound **3** (6.9 g, 28 mmol) was mixed with 20 mL anhydrous THF and added dropwise to the mixture of piperidine (2.4 g, 28 mmol), K_2CO_3 (7.7 g, 56 mmol) and anhydrous THF (40 mL) under N_2 at 0 °C. After vigorous stirring for 30 min, the ice bath was removed and the reaction mixture was kept stirring at room temperature for another 12 h. The reaction mixture was concentrated under the reduced pressure and redissolved in ethyl acetate (50 mL), followed by thorough washing with brine (5 × 50 mL). After drying over anhydrous $MgSO_4$, the organic layer were filtered and concentrated under reduced pressure to give a yellow residue, which was purified by flash column chromatography (SiO_2) eluting with mixed solvent of hexanes and ethyl acetate (hexanes : EtOAc = 3:1 v/v) to afford the titled compound **P-PA** as sticky yellow oil: 6.7 g (85%). ^{1}H NMR (500 MHz, d-DMSO, ppm): δ 7.80 (d, *J* = 7.5 Hz, 1H), 7.67 – 7.69 (m, 2H), 7.49 – 7.45 (m, 1H), 4.20 – 4.10 (m, 2H), 3.46 – 3.42 (m, 1H), 3.29-3.05 (br, 4H), 1.51 – 1.44 (m, 5H), 1.27 (d, *J* = 7.0 Hz, 3H); ^{13}C NMR (126 MHz, d-DMSO, ppm) δ 154.03, 150.34, 136.79, 132.77, 128.45, 126.76, 123.47, 68.52, 44.15, 33.09, 25.11, 23.72, 17.50. ESI-MS (m/z): $[M+Na]^+$ monoisotopic peak for C15H20N2O4Na, calculated: 315.1321, found: 315.1318; The isotopic signature observed for this compound also confirmed its elemental composition.

Supplementary Figures

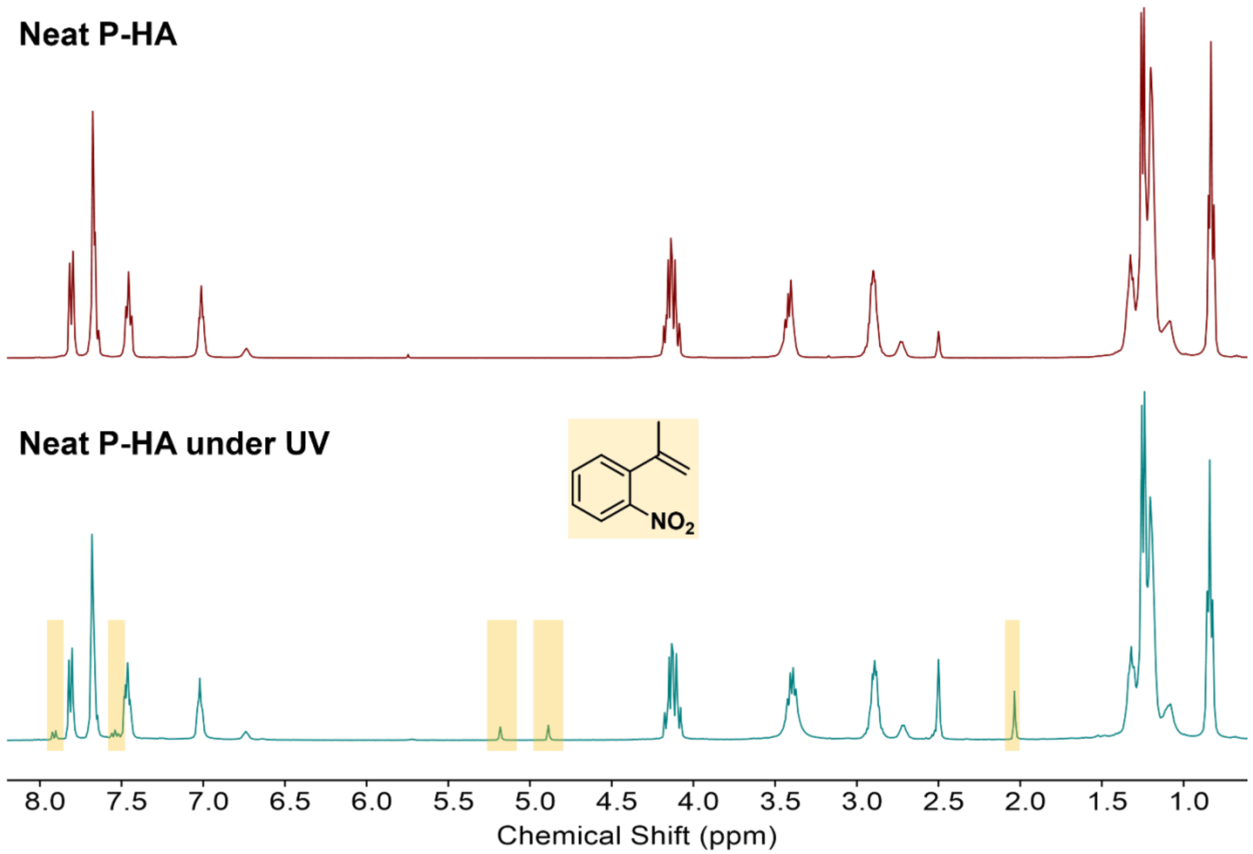

Supplementary Fig. 2 | ^{1}H NMR spectra (400 MHz, DMSO-*d*6) of neat P-HA before and after UV exposure (30 mW·cm^{-2}) for 1 h. 2-(2'-nitrophenyl)propene was the primary byproduct observed, confirming the UV-deprotection mechanism of NPPOC.

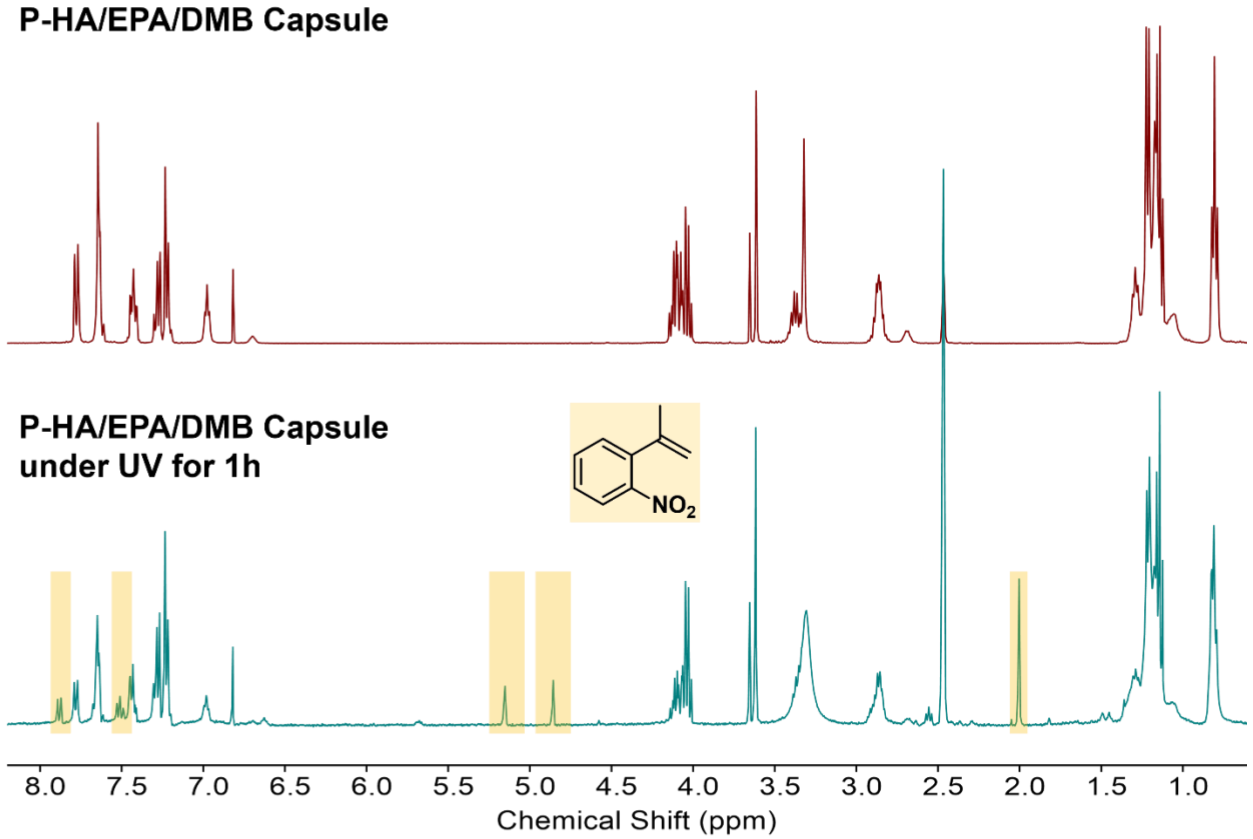

Supplementary Fig. 3 | ^{1}H NMR spectra (400 MHz, DMSO-*d*6) of P-HA extracted from microcapsules before and after UV exposure (30 mW·cm^{-2}) for 1 h. 2-(2'-nitrophenyl)propene was the primary byproduct observed, confirming the same UV-deprotection mechanism of NPPOC occurred within the microcapsules.

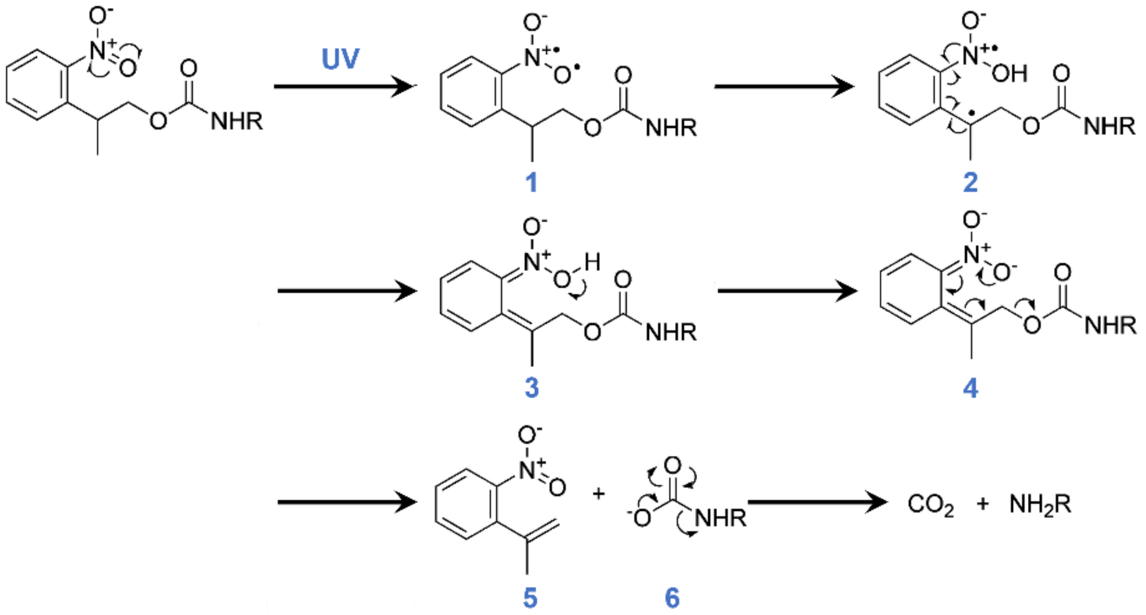

Supplementary Fig. 4 | The photolysis mechanism of NPPOC-protected amines. Intermediates with radicals and enhanced conjugations may form in the capsule payloads containing high amine content under intense UV irradiation.

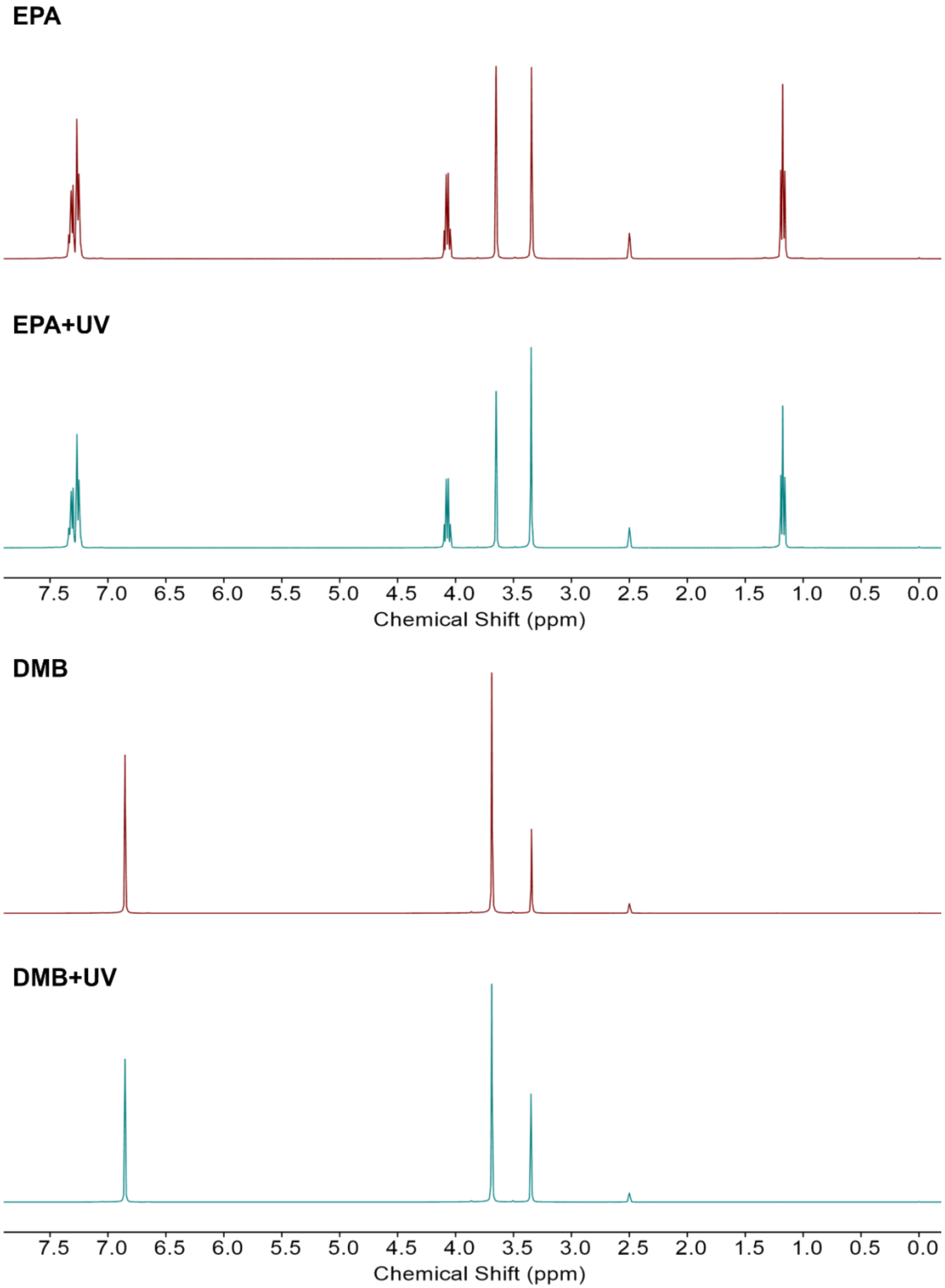

Supplementary Fig. 5 | ^{1}H NMR spectra (400 MHz, DMSO-*d*6) of pure EPA and DMB before and after UV exposure (30 mW·cm^{-2}) for 1 h. EPA and DMB showed no change upon UV radiation.

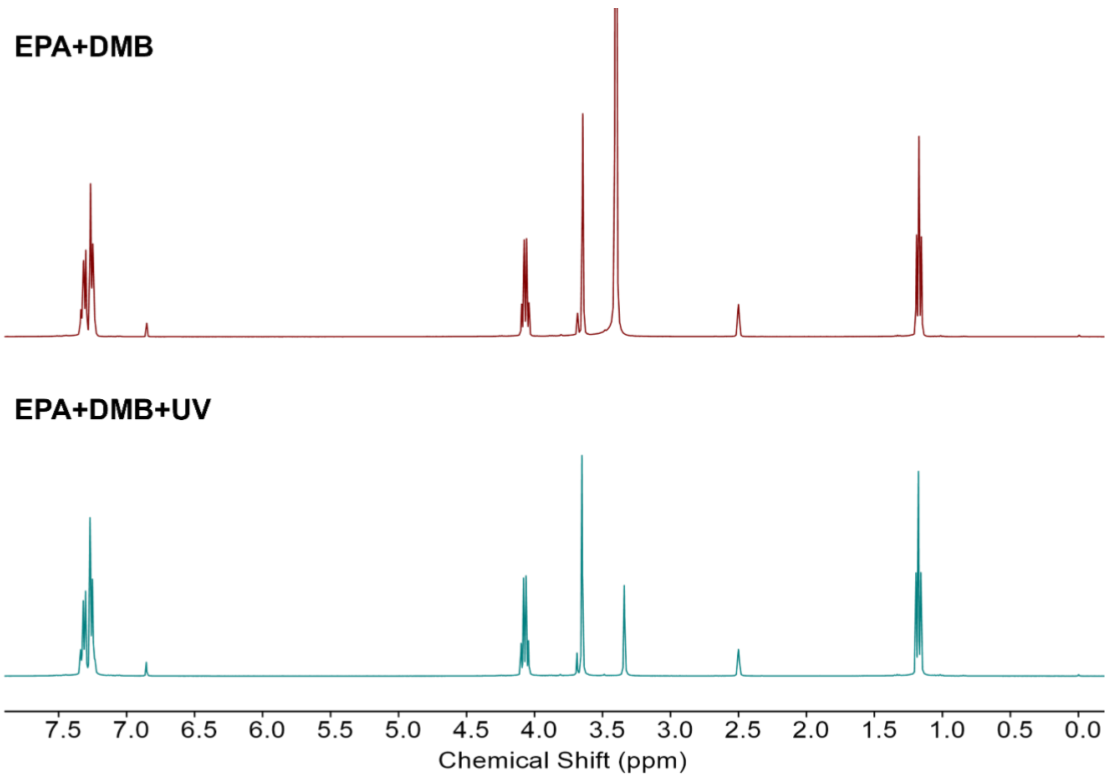

Supplementary Fig. 6 | ^{1}H NMR spectra (400 MHz, DMSO-*d*6) of a 2 wt% DMB in EPA solution before and after UV exposure (30 mW·cm^{-2}) for 1 h. The mixture showed no change upon UV radiation.

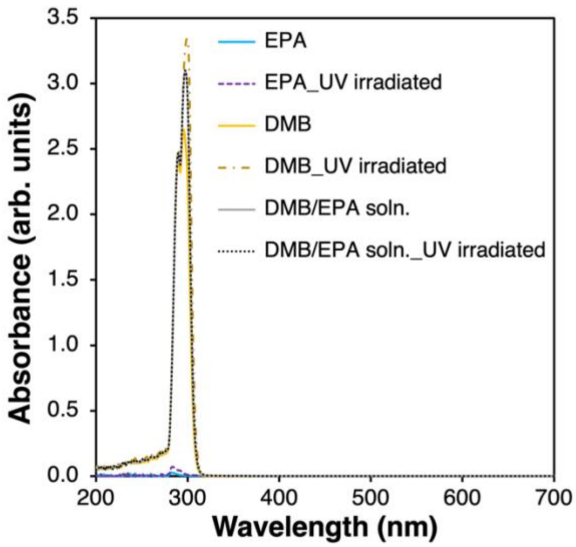

Supplementary Fig. 7 | Chemical and optical studies of the photolysis reaction. (a) UV-Vis spectra of EPA, DMB, and 2 wt% DMB in EPA before and after 1 h exposure to a 365 nm light at an intensity of 30 mW·cm^{-2}.

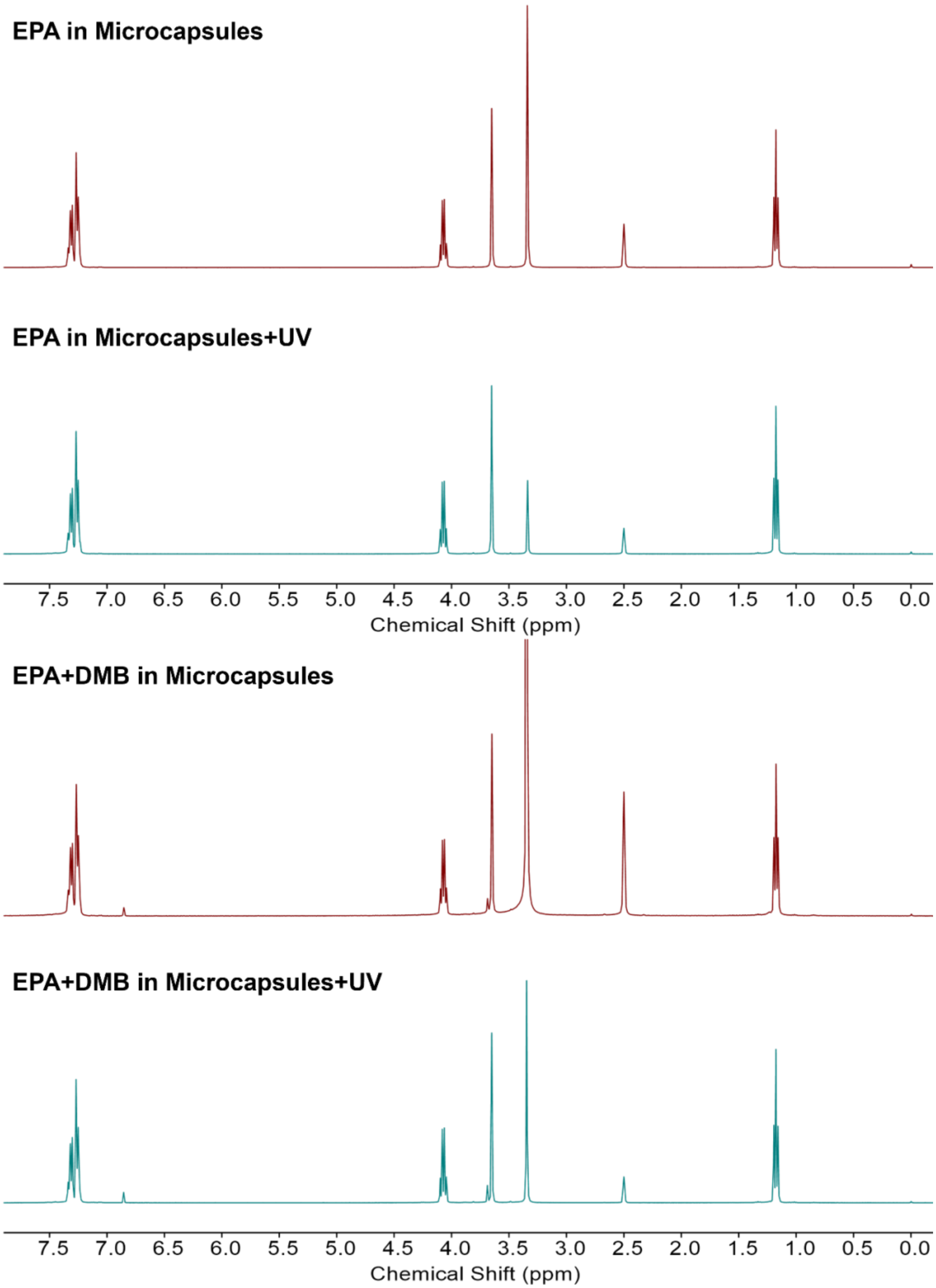

Supplementary Fig. 8 | ^{1}H NMR spectra (400 MHz, DMSO-*d*6) of EPA capsules and 2 wt% DMB in EPA capsules before and after UV exposure (30 mW·cm^{-2}) for 1 h. EPA and DMB showed no change upon UV radiation within microcapsules.

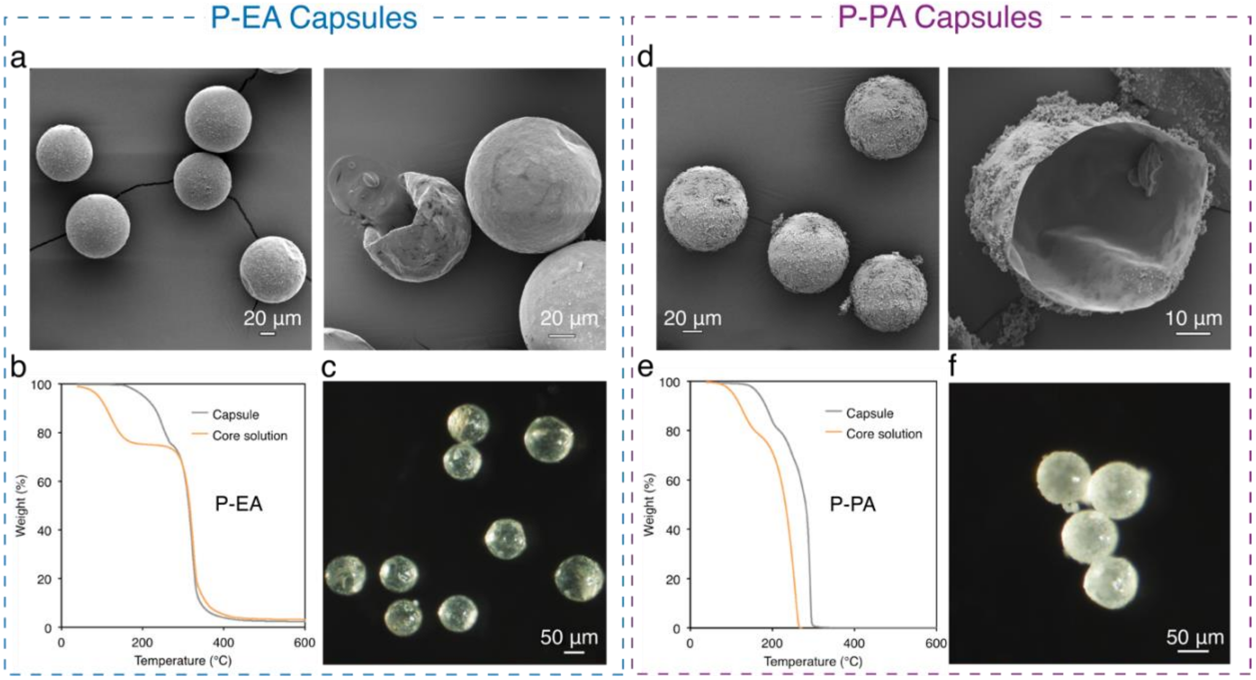

Supplementary Fig. 9 | Structural characterization of microcapsules containing 80 wt% protected amines and 20 wt% EPA. (a) SEM images of intact and ruptured P-EA capsules. (b) TGA of P-EA capsules demonstrates significantly enhanced thermal stability compared to the unencapsulated core solution. (c) Stereomicrograph of P-EA capsules showing a clear, off-white appearance. (d) SEM images of intact and ruptured P-PA capsules. (e) TGA of P-PA capsules demonstrates significantly enhanced thermal stability compared to the unencapsulated core solution. (f) Stereomicrograph of P-PA capsules showing a clear, off-white appearance. Each experiment was independently repeated three times, yielding consistent results.

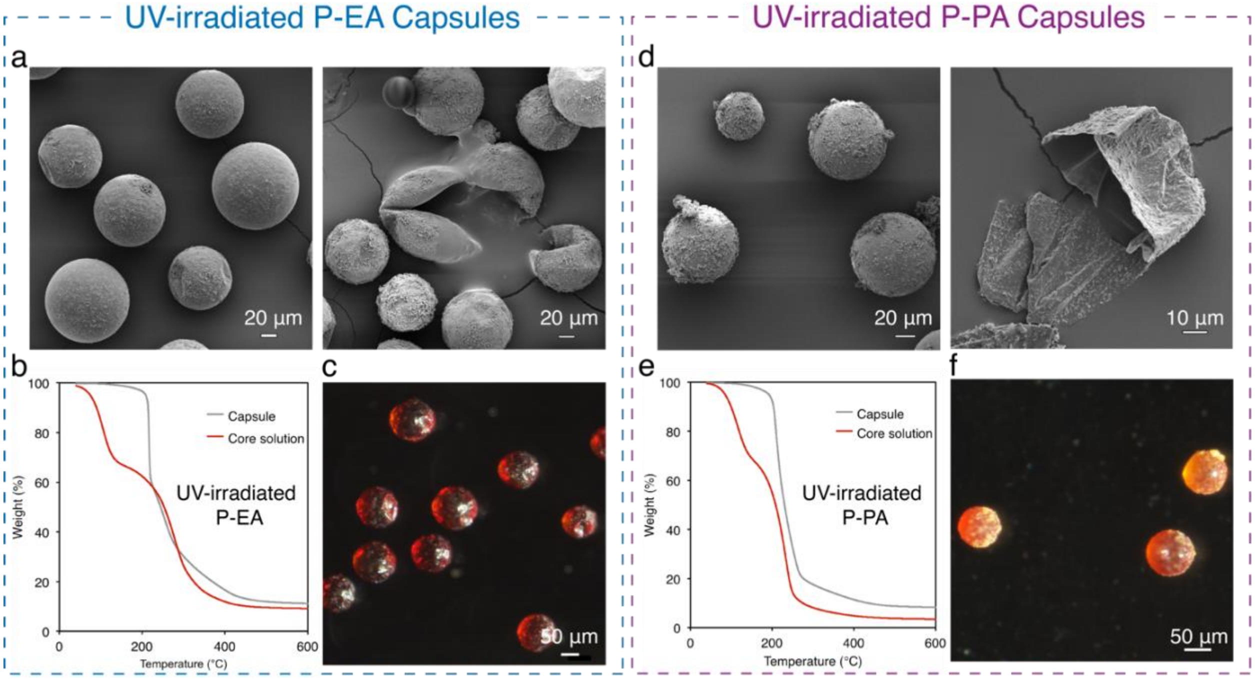

Supplementary Fig. 10 | Structural characterization of microcapsules containing 80 wt% protected amines and 20 wt% EPA after 1 h 365 nm UV exposure at 30 mW·cm^{-2}. (a) SEM images of intact and ruptured UV-irradiated P-EA capsules. (b) TGA of UV-irradiated P-EA capsules demonstrates the preservation of enhanced thermal stability compared to the unencapsulated, UV-irradiated core solution. (c) Stereomicrograph of UV-irradiated P-EA capsules showing a brown color. (d) SEM images of intact and ruptured UV-irradiated P-PA capsules. (e) TGA of UV-irradiated P-PA capsules demonstrates the preservation of enhanced thermal stability compared to the unencapsulated, UV-irradiated core solution. (f) Stereomicrograph of UV-irradiated P-PA capsules showing a brown color. Each experiment was independently repeated three times, yielding consistent results.

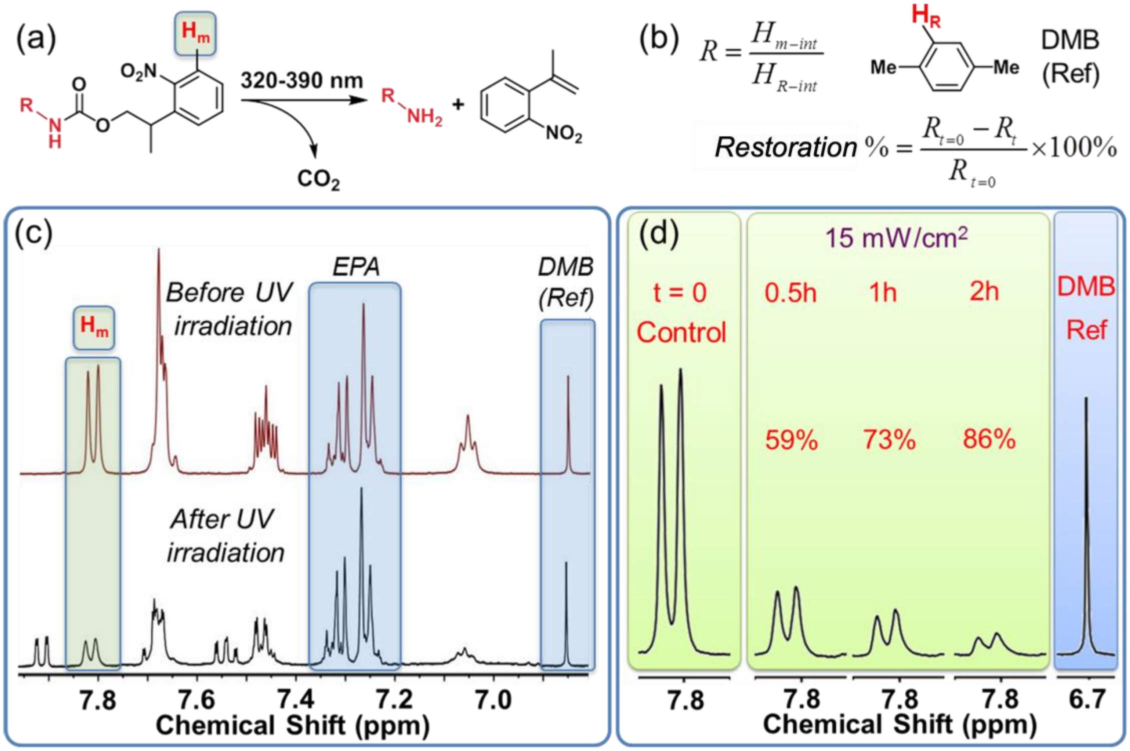

$$R = \frac{H_{m-int}}{H_{R-int}}$$

$$\text{Restoration } \% = \frac{R_{t=0} - R_t}{R_{t=0}} \times 100\%$$

Supplementary Fig. 11 | Methodology of photoactivation studies. (a) Photolysis reaction of NPPOC-protected amines under UV irradiation. The highlighted hydrogen was used as an NMR marker group for restoration rate calculations. (b) Calculation method to determine the restoration rate of masked amines of intact microcapsules. R is the ratio of NMR integration of H_m and H_R. *p*-Dimethylbenzene (DMB, 2 wt% in payloads) was used as a reference reagent. (c) Representative NMR spectra of P-EA before and after UV irradiation. (d) Normalized NMR peak intensities of H_m at different irradiation times and H_R.

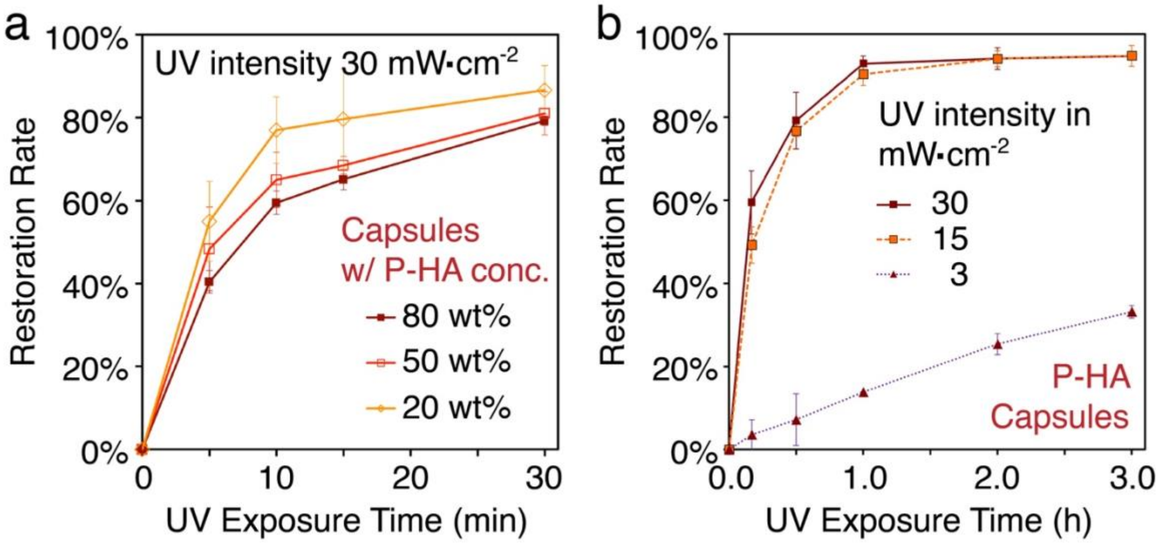

Supplementary Fig. 12 | Extended investigation into photolysis kinetics of P-HA capsules. (a) Time-dependent amine restoration in capsules with payloads of varying P-HA concentrations. (b) Photoactivation efficiency of capsules with 80 wt% P-HA under 365 nm UV light of different intensities. According to Air Mass 1.5 Spectra (ASTM G-173), the estimated UV intensity of sunlight (sum of wavelengths from 280 nm to 400 nm) is approximately 3 mW/cm^2. Data are presented as mean values ± SD (n = 3 independent samples).

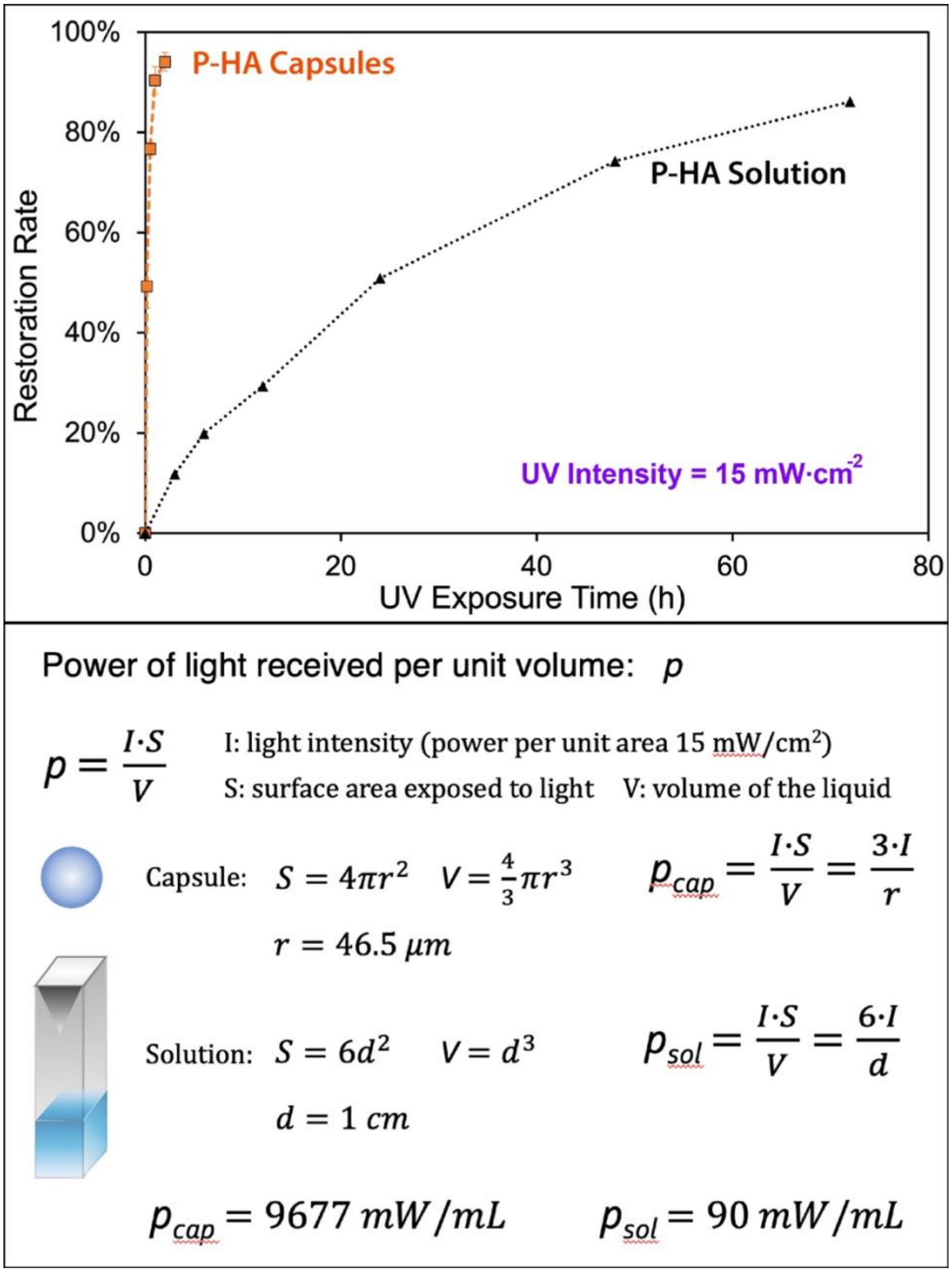

Supplementary Fig. 13 | Time-dependent amine restoration in capsules vs. in quartz cuvette. Both the capsules and the cuvette contain the same 80 wt% P-HA in EPA solution. A 365 nm UV light was applied at the intensity of 15 mW·cm^{-2}. Protected amines received approximately two orders of magnitude stronger UV light when retained within core-shell microcapsules.

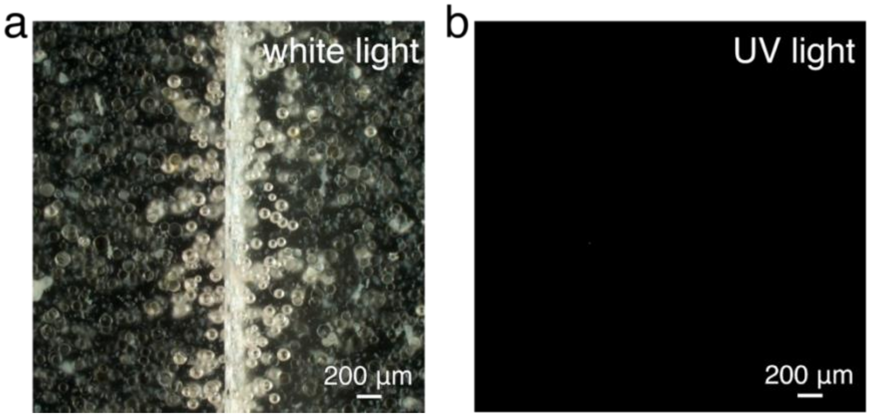

Supplementary Fig. 14 | Stereomicrographs of stylus-scratched PDMS coating containing 7.5 wt% NBD-Cl capsules and 2.5 wt% P-HA capsules under (a) white light and (b) UV light.

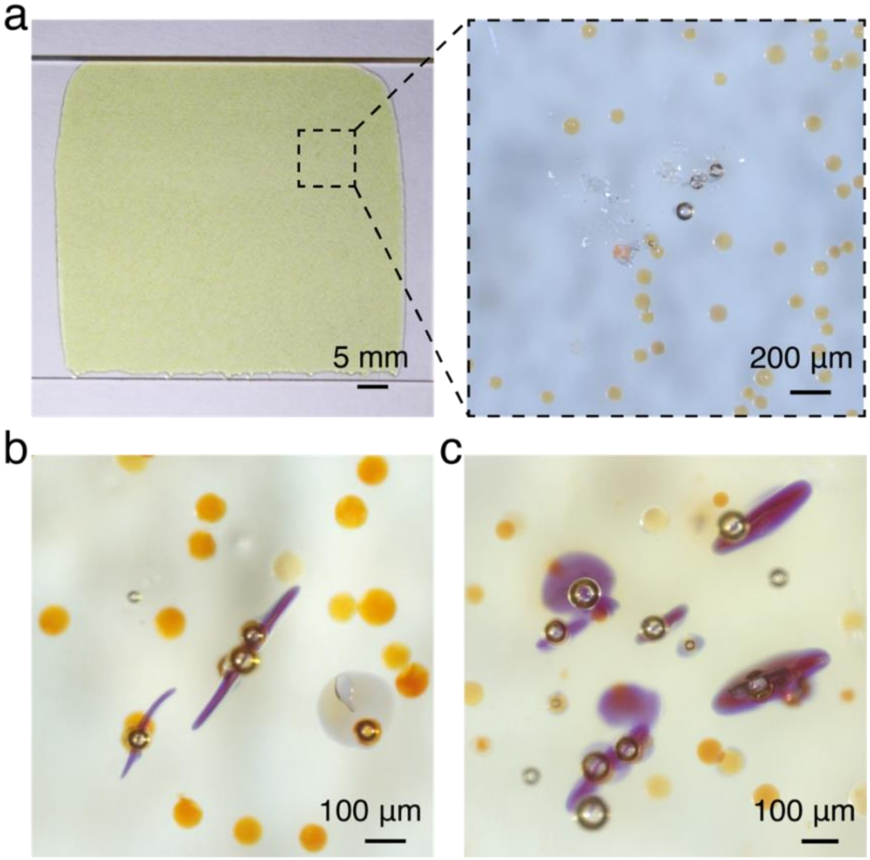

Supplementary Fig. 15 | (a) Photograph and stereomicrograph of a control epoxy specimen loaded with 2 wt% P-HA capsules. The focused ultrasound created internal damage that ruptured microcapsules. No color change was generated due to the lack of active amines. Stereomicrographs of ultrasound-impacted epoxy coatings containing (b) 2 wt% A-EA capsules and (c) 2 wt% A-PA capsules. 0.15 wt% BPB was dispersed in all specimens.

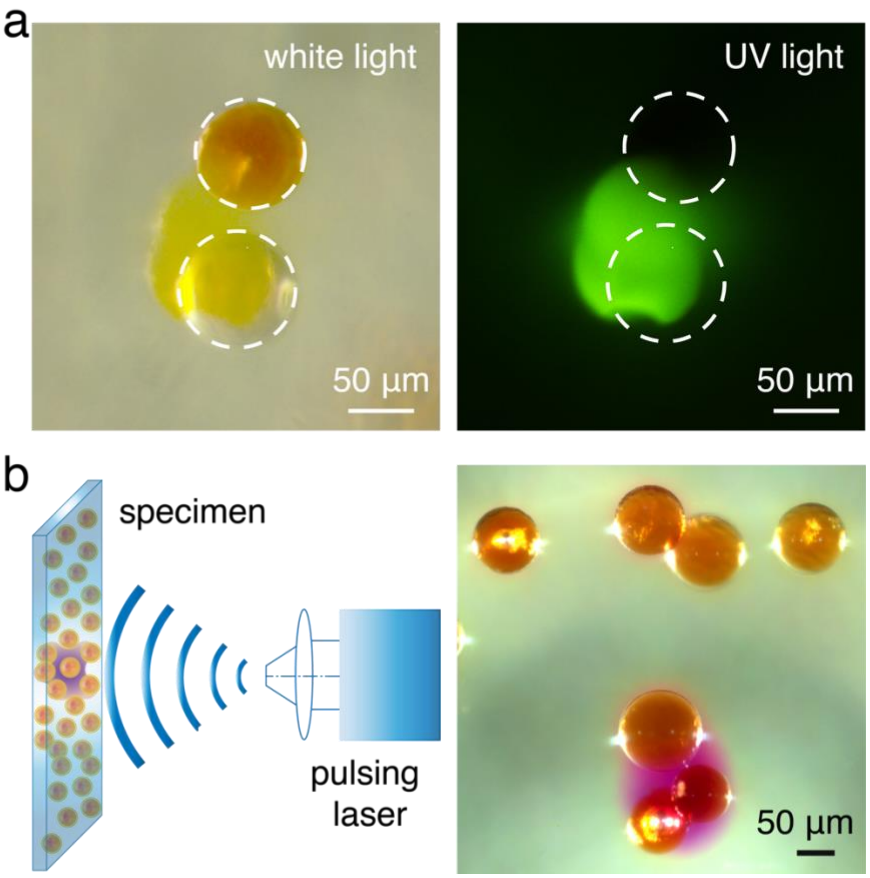

Supplementary Fig. 16 | (a) Stereomicrographs of A-HA capsules under illumination with white light and UV light illustrating autonomous reporting of ultrasound traces. Focused ultrasound was directed towards capsules suspended in an NBD-Cl solution, resulting in the emission of vivid green fluorescence that visually tracked the payload release. The locations of capsule shells are outlined as a guide (white dashed circles). (b) A laser-induced shockwave created internal damage within an epoxy specimen. The embedded A-HA capsules discharged active amines that activated the built-in BPB molecules and initiated a localized color indication.

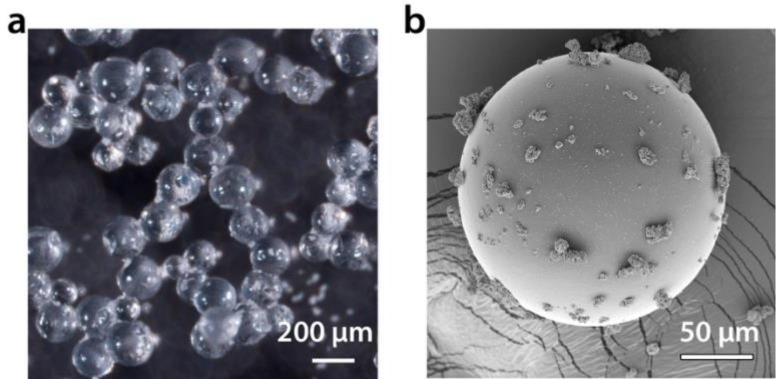

Supplementary Fig. 17 | Epoxy microcapsules. (a) Optical micrograph. (b) SEM image. Each experiment was independently repeated three times, yielding consistent results.

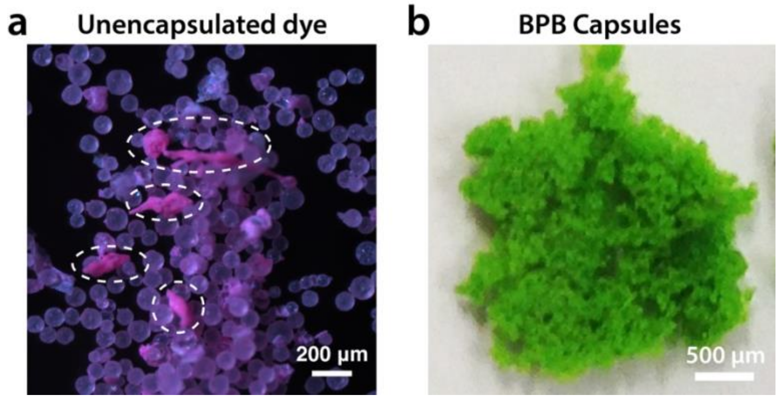

Supplementary Fig. 18 | Challenges with dye encapsulation. (a) Genacryl Pink G left outside of the core-shell microcapsules. Unencapsulated dyes are outlined with white dashed circles. (b) BPB microcapsules showing a strong green color.

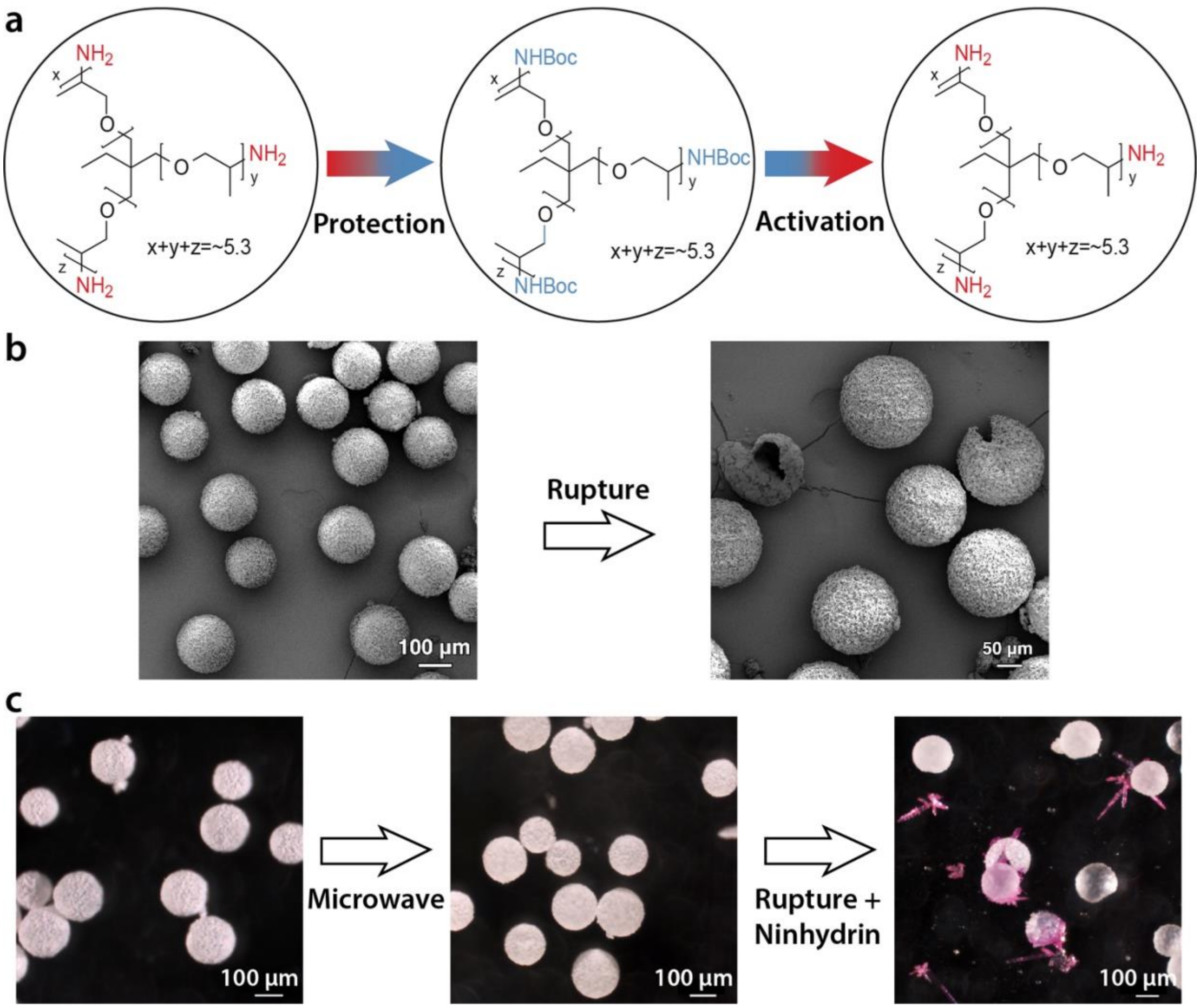

Supplementary Fig. 19 | Thermally modulated activation of amines for microencapsulation and on-demand reactivity. (a) Reactivity modulation of polyoxypropylene triamine (EPIKURE 3233) using BOC protecting group. (b) SEM micrographs of core-shell microcapsules containing protected-amines. (c) Amine restoration within intact microcapsules was achieved by microwave irradiation. Active amines were confirmed by the colorimetric reaction between mechanically released payload and a ninhydrin droplet. Each experiment was independently repeated three times, yielding consistent results.

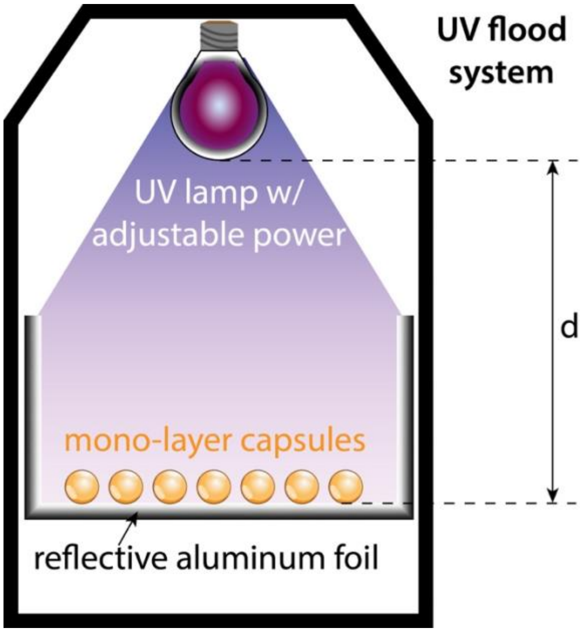

Supplementary Fig. 20 | Schematic of the photoactivation setup using a UV flood system. Microcapsules were evenly dispersed on a reflective aluminum tray, promoting a monolayer distribution. The tray was positioned in a UV flood system, where UV intensity was accurately controlled by adjusting power of the UV lamp and sample distance from the light source. Reflective aluminum foil was employed on the side walls to ensure 360° UV irradiation. The actual UV intensities were verified by a UV light meter.

Supplementary Tables

Supplementary Table 1. Viscosity evolutions of the capsule payloads upon 30 mW·cm^{-2} UV irradiation for 30 min. Measurements were conducted at a shear rate of 1 s^{-1} at 25 °C. Viscosity unit: Pa·s.

Payload	80 wt% P-HA in EPA	80 wt% P-EA in EPA	80 wt% P-PA in EPA
Original value	0.057	0.388	0.045
Irradiated value	0.072	0.993	0.112

Supplementary Table 2. Shear strength characterized by lap shear measurements. Values reported are maximum adhesive strength achieved at respective temperatures. Specimens using capsules were produced by crushing a combination of A-EA microcapsules and epoxy microcapsules at a 1-1 ratio by weight between glass slides. The control specimens were fabricated by spreading a blended pristine EA, epoxy, and EPA between glass slides. Shear strength of super glue was also examined for comparison. All specimens were cured at designated temperatures.

Reaction temperature	Shear strength induced by capsules (MPa)	Shear strength by pristine reagents (MPa)	Ratio of capsule vs. pristine
20 °C	0.55	0.85	64.7%
35 °C	1.46	2.18	67.0%
50 °C	2.00	2.77	72.2%

Note: maximum shear strength achieved by super glue was 2.76 MPa.

Supplementary Reference

1. Bhushan, K. R., DeLisi, C. & Laursen, R. A. Synthesis of photolabile 2-(2-nitrophenyl)propyloxycarbonyl protected amino acids. *Tetrahedron Lett.* **44**, 8585-8588 (2003).
